# Research on the Temperature Characteristics of the Photoacoustic Sensor of Glucose Solution

**DOI:** 10.3390/s18124323

**Published:** 2018-12-07

**Authors:** Wei Tao, Zhiqian Lu, Qiaozhi He, Pengfei Lv, Qian Wang, Hui Zhao

**Affiliations:** Department of Instrument Science and Engineering, Shanghai Jiao Tong University, 800 Dongchuan Rd., Shanghai 200240, China; taowei@sjtu.edu.cn (W.T.); lzq1993@sjtu.edu.cn (Z.L.); heqz1993@sjtu.edu.cn (Q.H.); 116035910014@sjtu.edu.cn (P.L.); qianwangVera@sjtu.edu.cn (Q.W.)

**Keywords:** photoacoustic sensor, glucose solution, temperature coefficient, Grüneisen parameter

## Abstract

In order to weaken the influence of temperature on photoacoustic (PA) measurements and compensate PA signals with a proposed theoretical model, the relationship of PA signal amplitude with temperature, under the condition of different glucose concentrations and different light intensities, was studied in this paper. First, the theoretical model was derived from the theory of the PA effect. Then, the temperature characteristics of the PA signals were investigated, based on the analyses of the temperature-dependent Grüneisen parameter in glucose solution. Next, the concept of a PA temperature coefficient was proposed in this paper. The result of the theoretical analysis shows that this coefficient is linear to light intensity and irrelevant to the concentration of glucose solution. Furthermore, a new concept of a PA temperature coefficient of unit light intensity was proposed in this paper. This coefficient is approximately constant, with different light intensities and solution concentrations, which is similar to the thermal expansion coefficient. After calculation, the PA temperature coefficient by the unit light intensity of glucose solution is about 0.936 bar/K. Finally, relevant experiments were carried out to verify the theoretical analysis, and the PA temperature coefficient of the unit light intensity of glucose solution is about 0.04/°C. This method can also be used in sensors measuring concentrations in other aqueous solutions.

## 1. Introduction

At present, diabetes mellitus, one of the most common chronic diseases, seriously threatens human health. According to the statistics from International Diabetes Federation, the number of diabetic patients worldwide will reach 629 million by 2045 [1]. The blood glucose concentration of diabetic patients fluctuates significantly in one day, and the possible consequences include renal failure, apoplexy, hypertension, blindness and coma [2]. For diabetic patients, a timely and appropriate adjustment of the insulin dosage, based on the self-monitoring of blood glucose (SMBG) levels, is an effective and essential treatment [3,4]. It is important for SMBG results to be accurate and precise [5]. A glucose sensor plays a key role in the accurate measurement of blood glucose. There are mini-invasive glucose sensors and non-invasive sensors for SMBG, and non-invasive techniques have been a research hotspot due to their high sensitivity and better patient compliance, compared to invasive ones [6].

Non-invasive glucose sensors are based on various techniques including optical and non-optical ones. Optical methods include absorption, Raman, fluorescence and photoacoustic (PA) spectroscopy. Non-optical methods mainly include the electrochemical sensor method, energy conservation method, etc. [7]. Among them, PA spectroscopy is the most promising non-invasive measurement method of blood glucose, because of its high sensitivity to weakly absorbed substances, low optical reflection and scattering coefficient [8]. Therefore, glucose sensors based on the PA effect can become the most promising technique for advanced SMBG.

However, temperature strongly affects the performance of PA-based glucose sensors, because the amplitude of the PA signal has a strong correlation with temperature [9]. This could be due to the changes of sound velocity in the solution, density of materials, coefficient of absorption, etc. From in vivo measurements, the body temperature has been found to vary among patients. Furthermore, the patient’s body temperature shows a notable change in one day [10]. Therefore, it is necessary to weaken the interference of the temperature in order to improve the performance of PA-based glucose sensors.

The photothermal issue has been studied in various areas, such as in thin metal films, nanoparticles, nanoscale chemical and subsurface mapping of materials [11,12,13,14,15], and the relationship between temperature and PA signals has been studied in several papers [16,17,18,19,20,21,22,23]. Kottmann et al. studied the method of PA measurement for glucose in human skin tissue based on quantum cascade laser. They recorded the temperature in the PA cell by a temperature sensor to control the variations and improve the accuracy of results [24]. Tanaka et al. proposed a method called differential PA spectroscopy to overcome the scattering in measurement. They demonstrated that the change of temperature could make an impact on the PA signals and investigated the sensitivity of signals under the condition of varied temperature from 30 °C to 40 °C [25]. It was demonstrated that the dependency of the PA signal amplitude on temperature is mainly attributed to the Grüneisen parameter [17]. Therefore, the Grüneisen parameter must be researched in order to obtain greater knowledge of the effect of temperature on PA signals.

Recently, there have been several research groups focusing on the Grüneisen parameter in the PA effect. Mahmoodkalayeh et al. studied the PA noise and temperature based on a temperature-dependent parameter, called the Grüneisen parameter in the PA effect. They obtained the equations between PA noise and temperature and drew a conclusion that decreasing the temperature of the intermediated medium should improve the signal-to-noise ratio (SNR) of PA signals. They performed a simulation study with different temperatures and an experiment with a water phantom and a chicken breast sample. The results supported their conclusion that the SNR of PA signals was improved by lowering the medium temperature [26]. Yao et al. measured the Grüneisen parameter at room temperature by PA spectroscopy. The results demonstrated that this parameter of lipid, fat tissue and serum was constant at room temperature. Moreover, the Grüneisen parameter of red blood cells showed a good linear relationship with the concentration [27]. Liang et al. investigated the PA method to estimate the Grüneisen parameter of lipids. The researchers applied frequency-domain photoacoustics to obtain the fitted polynomial between the Grüneisen parameter and temperature [28]. The PA temperature characteristics are mainly dependent on the Grüneisen parameter. However, there are few studies that combine the concentration and the temperature to study the Grüneisen parameter of substances, especially in glucose aqueous solutions. There is no sufficient evidence that demonstrates the relation of the Grüneisen parameter to the temperature with varied concentrations of glucose. 

In this paper, the temperature characteristics of the PA signal were studied in detail by establishing a complete mathematical model between the Grüneisen parameter and temperature. A new concept of a PA temperature coefficient was proposed, and the PA temperature coefficient was calculated by the analysis results. The method of weakening the influence of temperature on the photoacoustic measurement by the PA temperature coefficient of unit light intensity was proposed. Finally, the conclusion has been verified by experiments.

## 2. Temperature Characteristics in the PA Measurement

### 2.1. Theoretical Model

According to the basic theory of the PA effect, the temperature of a medium is increased by photoabsorption, and this decreases while the light source is non-luminous. With a modulated laser, this periodic change results in the synchronous periodic expansion and contraction of the matter, which produces PA pressure waves. The amplitude of the PA pressure wave is closely related to the constituent of the medium [29].

According to the study by Patel et al. [30], the PA pressure wave, generated in a solution and excited by the pulsed laser, can be expressed as:

(1) $$P=(\frac{1}{\pi dR^{2}})\left(\frac{\beta \nu^{2}}{C_{P}}\right)E\alpha .$$

where *p* represents the amplitude of the PA pressure, *R* represents the diameter of the laser beam, *ν* is the speed of sound in the solution, *E* is the energy of the pulsed laser, *d* represents the vertical distance from the PA sensor to the axis of laser beam, *β* represents the coefficient of thermal expansion, *C_p_* is the specific heat capacity, and *α* is the absorption coefficient of the medium.

Obviously, the PA signal amplitude *P* is linearly related to the laser energy *E*, in accordance with energy conservation. In the process of measurement, the laser beam diameter *R* and the vertical distance *d* also affect the amplitude *P* of the PA signal. The laser energy density is positively correlated with the diameter *d* of the laser beam, which determines the amplitude of the PA signal. A stronger PA signal can be achieved by shortening the vertical distance between the PA sensor and laser beam.

Considering a fixed PA measurement system, the laser beam diameter *R* and the vertical distance *d* are fixed parameters. Then, the formula *F* = *E*/(*πdR*^2^) can be used to represent the laser energy density, and the PA pressure wave can be simplified to the following equation:

(2) $$P=(\frac{\beta \nu^{2}}{C_{P}})\alpha F.$$

In Equation (2), we use *Г* to represent the term *βν*^2^/*C_p_*, so we can know that:

(3) $$Г=\frac{\beta \nu^{2}}{C_{p}}$$

where *Г* is the Grüneisen parameter, mentioned above. Considering the fact that the relationship between PA signals and the temperature mainly depends on this parameter, we can clarify this by the following equation:

(4) P(T)=αFГ(T)

### 2.2. Temperature Characteristics of the Photoacoustic (PA) Signals

Based on the research of Mackenzie et al. [31], the absorption coefficient of glucose solution almost remains constant with various concentrations at a wavelength of 1064 nm. According to the results of Curcio and Petty [32], the absorption coefficient of water at 1064 nm is 0.013 mm^−1^.

Referring to Equations (3) and (4), we should investigate the main parameters of the Grüneisen coefficient to clearly illustrate the PA temperature characteristics. The relationship among these primary factors (thermal expansion coefficient, specific heat capacity and sound velocity), concentration of glucose and temperature is illustrated as follows.

(1) The relationship among the thermal expansion coefficient, temperature, and concentration:

Referring to the study of Darros-Barbosa et al. [33], the relationship among the thermal expansion coefficient *β*, solution density *ρ*, and temperature *T* is (at the standard atmospheric pressure):

(5) $$\beta =-\frac{1}{\rho }\left(\frac{\partial \rho }{\partial T}\right).$$

Obviously, the thermal expansion coefficient *β* is directly related to the density *ρ* and the temperature of liquid *T*.

Darros-Barbosa measures the density ρ of glucose aqueous solution with a temperature of between 5 °C and 65 °C at the standard atmospheric pressure to obtain an approximate polynomial of density *ρ*, concentration *C* (g/dL) and temperature *T* (°C):

(6) $$\rho =(x_{1}+y_{1}C+z_{1}C^{2})+T(x_{2}+y_{2}C+z_{2}C^{2})+T^{2}(x_{3}+y_{3}C+z_{3}C^{2}).$$

where *x_i_*~*z_i_* are the regression coefficients, and the values are shown in Table 1.

Equation (7) is derived from Equations (5) and (6):

(7) $$\beta =\frac{\sum_{i=1}^{2}\sum_{j=2}^{3}i(x_{j}+y_{j}C+z_{j}C^{2})T^{i-1}}{\sum_{i=1}^{3}\sum_{j=1}^{3}(x_{j}+y_{j}+z_{j}C^{2})T^{i-1}}.$$

Obviously, the thermal expansion coefficient *β* is related to the density *ρ*, concentration *C*, and temperature *T*.

(2) The relationship among the sound velocity, temperature, and concentration:

Contreras [34] conducted an experiment to analyze the sugar contents in beverages, giving an approximate polynomial of sound velocity *ν*, solution concentration *C* (unit is percentage *w*/*v*) and temperature *T* (unit is °C) in the solution of glucose:

(8) $$v=a_{1}+a_{2}C+a_{3}T+a_{4}C^{2}+a_{5}T^{2}+a_{6}CT.$$

where *a*_1_~*a*_6_ are the regression coefficients, and the approximate values given by the experiments are *a*_1_ = 1407.0, *a*_2_ = 4.41, *a*_3_ = 4.35, *a*_4_ = 0.0151, *a*_5_ = −0.0311, *a*_6_ = −0.0371.

This indicates that the sound velocity *ν* is only related to the concentration *C* and the temperature *T*.

(3) The relationship among the specific heat capacity, temperature, and concentration:

According to the research results of binary solutions by Darros-Barbosa [35], the specific heat capacity of any binary solution can also be expressed by a polynomial of concentration *C* (kg/m^3^) and temperature *T* (K):

(9) $$C_{\rho }=\sum_{i=1}^{3}(\sum_{j=0}^{2}b_{ij}C^{j})T^{i-1}.$$

In the formula, *b_ij_* are the regression parameters for the glucose solution, and the values are shown in Table 2.

(4) Comprehensive analyses:

By substituting Equations (7)–(9) into Equation (2), the amplitude of the PA pressure signal can be obtained:

(10) $$P=[2.5C+0.005C^{2}-(27+0.07C)T+0.16T^{2}+1329]\alpha F.$$

This indicates that the amplitude *P* (unit is bar) of the PA pressure signal is related to the concentration *C* (unit is kg/m^3^), temperature *T* (unit is K)*,* and light intensity *F* (unit is J), and the effects of these parameters are not separated. In order to quantitatively analyze the influence of temperature on the PA signal, the concept of a PA temperature coefficient was proposed in his paper. This coefficient can be understood as the change of the PA pressure signal caused by a unit temperature change, which is:

(11) k=∂p/∂T.

Substituting Equation (10) for Equation (11), the partial differential of the amplitude *P* at the temperature *T* gives the PA temperature coefficient:

(12) $$k=\frac{\partial P}{\partial T}=(0.32T-0.07C-27)\alpha F.$$

For human blood glucose measurement, the range of the concentration *C* is 0–2 kg/m^3^, and the temperature *T* is between 303 K and 313 K. According to Equation (11), the influence of the concentration *C* on the PA temperature coefficient is much smaller than that of the temperature *T*, which is negligible. Thus, *k* can be approximated as:

(13) $$k=\frac{\partial P}{\partial T}\approx (0.32T-27)\alpha F.$$

Since the relative change in the temperature *T* ranges from 303 K–313 K, which is only a 3% change, we assume that the value is 310 K. Therefore, the PA temperature coefficient can be approximated as:

(14) k=0.936F.

According to Equation (13), the PA signal has a good linear correlation with temperature. The PA temperature coefficient *k* is only related to the light intensity *F*. In order to eliminate the influence of light intensity, a new concept of a PA temperature coefficient of unit light intensity was proposed in this paper, which is approximately constant (unit is bar/K), with different light intensities and solution concentrations, which is similar to the thermal expansion coefficient. According to Equation (14), the PA temperature coefficient of the unit light intensity of glucose solution is k* = 0.936 bar/K.

This coefficient can be used to eliminate the effect of temperature on the PA signal and improve the measurement accuracy. This concept can also be applied to concentration measurements of other aqueous liquids.

## 3. Experiment

### 3.1. Experiment System and Equipment

**Figure 1:**
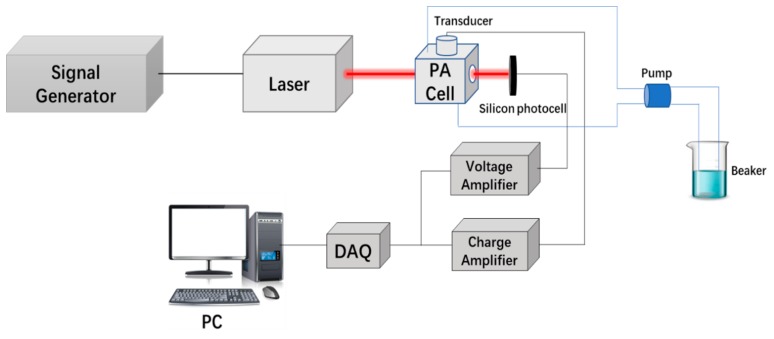
The framework of the photoacoustic (PA) measuring system.

**Figure 2:**
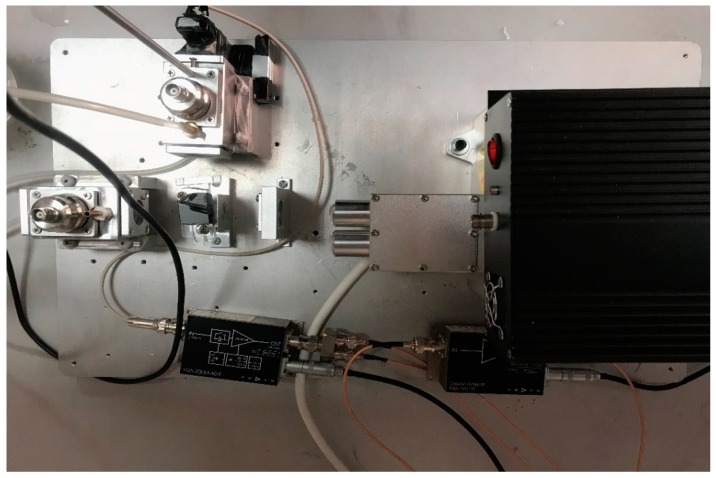
The experiment setup of the PA measuring system.

The structure of the experimental system is shown in Figure 1. A function generator (AFG310, Tektronix Japan, Ltd., Tokyo, Japan) was used to generate the trigger signal, which was simultaneously sent to the driver of the Nd:YAG (STL1064QW-1 mJ, Stone Laser Co., Ltd., Beijing, China) laser and high-speed data acquisition card (DAQ, Spectrum M2i.202, Bielefeld, Germany). The excitation wavelength of the laser was 1064 nm. The width of the laser pulse was 9.2 ns, and the repetition frequency was 100 Hz. The PA cell was full of glucose solution. The customized transducer (PZT-5A, 0.5 MHz center frequency, cylindrical with 25 mm diameter) was employed to convert the PA pressure wave into an electrical signal. Then, a charge amplifier (HQA-15M-10T, FEMTO^®^ Messtechnik GmbH, Schorndorf, Germany), with a gain of 10 V/pC, was used to amplify the PA signal, and it was sent to DAQ and recorded by a computer. The silicon photocell was used to record the amplitude of the light intensity of the laser, and a voltage amplifier (HVA-200M-40-F, FEMTO^®^ Messtechnik GmbH, Schorndorf, Germany) is used to amplify the output. The experiment setup is shown in Figure 2.

### 3.2. PA Signal and Temperature Test with Different Intensities of Light

The experiment was performed to investigate the variation of PA signals with different light intensities and solution temperatures. The testing sample was deionized water. At first, the laser was preheated for 1 h to keep it stable, and the intensity of the laser was adjusted to a proper level. The sample in the beaker was slowly filled into the PA cell by an electrical pump, until the liquid was fully mixed. Then, the liquid in the beaker was heated to 40 °C using an electrical heater. While the solution cooled down, the PA signal and the light intensity were synchronously acquired. As the output laser intensity was gradually increased from the minimum level 1 to the maximum level 6, all sampling data were recorded by DAC, including the PA pressure signal (V), the light intensity (V) and the temperature of the solution (°C).

**Figure 3:**
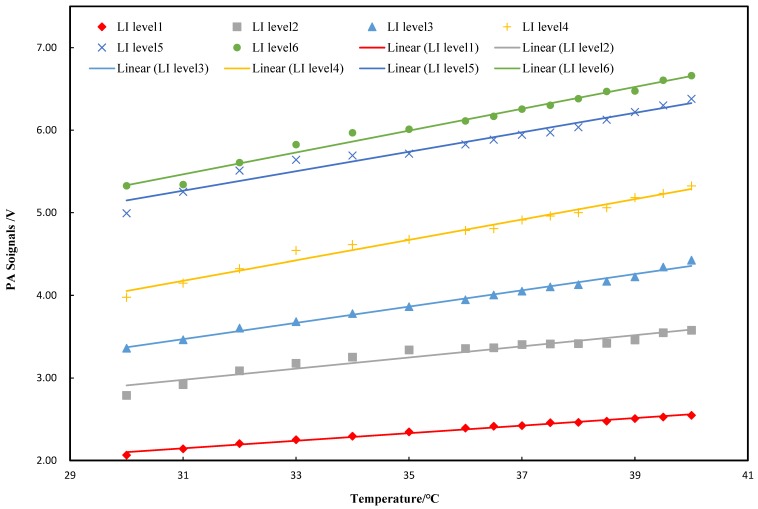
Relation of the PA signals to temperature at the different light intensities.

The relationship between the PA signal amplitude and temperature with the light intensity is shown in Figure 3. From the test results, the PA signal amplitude showed a good linear correlation with temperature (R^2^ = 0.99), which was consistent with the above conclusions.

The PA temperature coefficient (unit is V/°C) and light intensity(V) are shown in Table 3. This showed that the PA temperature coefficient increased from 0.046 V/°C to 0.13 V/°C, while the light intensity increased from level 1 to level 6.

**Figure 4:**
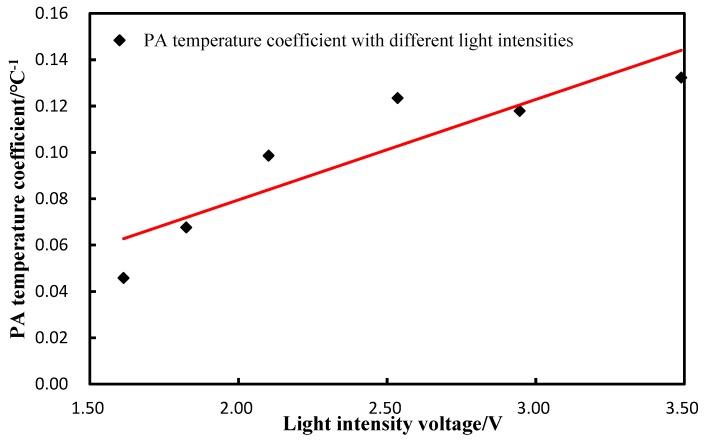
Relation of the temperature coefficient to light intensity.

The PA temperature coefficient, with different light intensities, which also showed good linearity and was completely consistent with the previous theoretical analyses, is plotted and shown in Figure 4.

### 3.3. Experiment of PA Temperature Characteristics with Different Concentrations

To determine the variation of the PA temperature characteristics induced by variable concentrations, an experiment of the temperature characteristic with different solution concentrations was performed. By keeping the laser light intensity fixed and changing the glucose concentration, the amplitude of the PA signal and temperature were measured.

**Figure 5:**
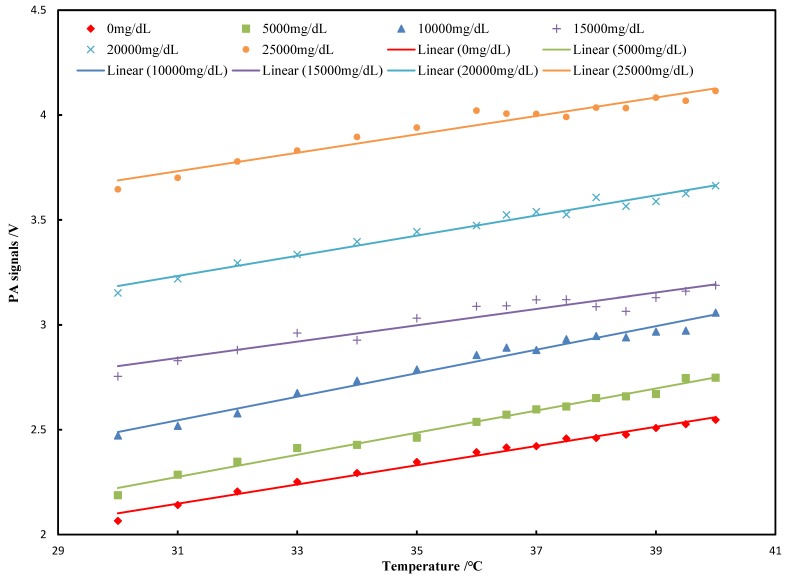
Relation of the PA signals to temperature under different concentrations.

The result of the experiment is shown in Figure 5. The PA signal still showed a good linear relationship with glucose concentrations (R^2^ = 0.99), which was consistent with the previous theoretical analyses.

**Figure 6:**
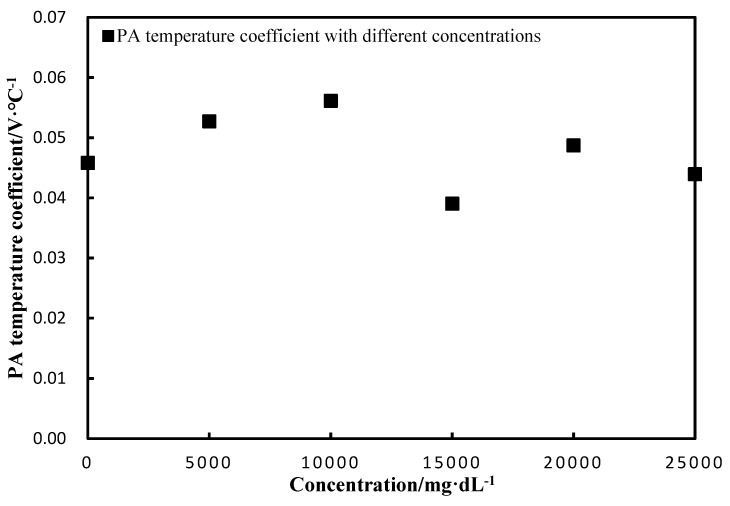
PA temperature coefficient with the concentration.

The PA temperature coefficient was weakly related to the concentrations, as shown in Figure 6. 

## 4. Discussion

The PA temperature coefficient is proposed in this paper to compensate for the deviation of the PA signal amplitude caused by the temperature. Our work focuses on the temperature-dependent coefficient, called the Grüneisen parameter. In several papers, this parameter in water was represented by the following form [36,37,38]:

(15) Г=A+BT

where *A* and *B* are constants. Therefore, the Grüneisen parameter is linear to the medium temperature. However, this linearity may change with varied concentrations of aqueous solutions, such as glucose solution. Therefore, our research starts with the relation between three factors of the Grüneisen parameter and temperature. We obtain the fitted polynomial of these factors and the polynomial of the PA signal amplitude with the temperature and the concentration. The temperature coefficient is calculated and measured by the experiments. The result in Figure 3 shows that the PA signals are linear with the temperature at different light intensities. It can be seen, from Figure 3, that the slopes increase with higher light intensities. These slopes represent the PA temperature coefficients and show good linearity with the light intensities, in accordance with Equation (12), as shown in Figure 4. The results on the PA signals with varied concentrations indicate that the slopes are almost constant. The correlation coefficient between them is −0.3, which means that changing the solution concentrations could not significantly affect the PA temperature coefficient, which is consistent with the result of Equation (13). This result demonstrates that the relationship between the Grüneisen parameter and temperature is still coincident with the linear relation with different glucose concentrations. Therefore, we clearly demonstrate the temperature characteristics of the PA signals with varied light intensities and glucose concentrations. 

**Figure 7:**
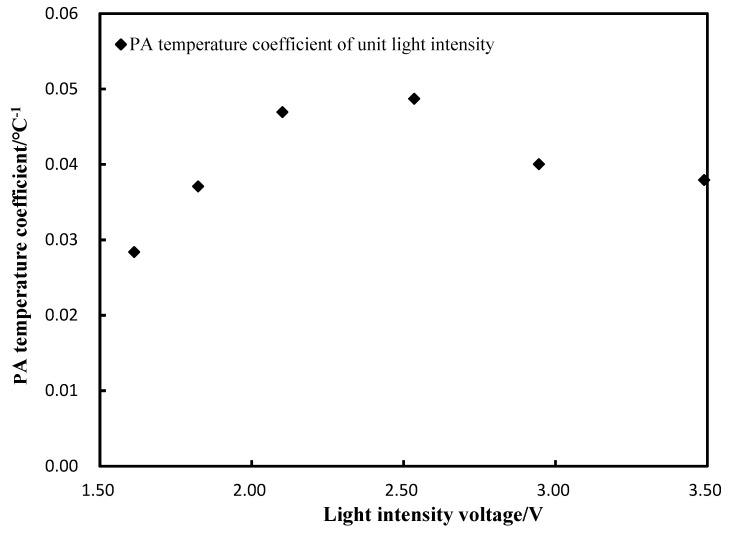
PA temperature coefficient of unit light intensity.

The PA temperature coefficient of unit light intensity (unit is °C^−1^) can be obtained by dividing the PA temperature coefficient by the light intensities, which is shown in Figure 7. It can be found that the temperature coefficient showed a weak correlation with light intensity (R^2^ = 0.29), which means it can be approximated as a constant (average value: 0.04/°C). This coefficient could be used to compensate for the amplitude of the PA signals with different temperatures of glucose solutions.

The method of using the PA temperature coefficient to compensate for the PA signals can be extended to other kind of solutions. Petrova et al. investigated the method for measuring the Grüneisen parameter in aqueous cupric sulfate solutions [39]. They found that the Grüneisen-temperature relationship shows a linear trend with the concentrations of cupric sulfate. In their work, they measured the optoacoustic intensity of CuSO_4_·5H_2_O, as a function of the temperature, with different concentrations. They obtained an experimental equation for the Grüneisen parameter, as a function of the temperature and concentration:

(16) $$Г(C,T)=\frac{\partial Г}{\partial T}(C)\cdot (T-T_{0}(C))$$

They did not conduct an experiment with different light intensities, although they demonstrated that the temperature dependency of the Grüneisen parameter of the most diluted salt solutions completely matched the result in water. Therefore, we can still use the following equations to represent the Grüneisen parameter of CuSO_4_·5H_2_O with an invariable concentration:

(17) $${Г}_{Cu}=A_{Cu}+B_{Cu}T$$

where *A_Cu_* and *B_Cu_* are both constants. Furthermore, we can still obtain the PA temperature coefficient of CuSO_4_·5H_2_O:

(18) $$k_{Cu}=\frac{\partial P_{Cu}}{\partial T}=\alpha B_{Cu}F$$

Therefore, the PA temperature coefficient is still effective in CuSO_4_·5H_2_O solution. The difference is that the Grüneisen-temperature relationship is irrelevant for concentrations of glucose solutions, according to our study. If we apply our method to compensate for CuSO_4_·5H_2_O solution, we should extend our PA temperature coefficient of unit light intensity to “PA temperature coefficient of unit light intensity and concentration”. The process and principle are the same. Therefore, this method should be effective for the temperature compensation of the PA signal amplitude in other solutions. The compensation process is as follows:

1. Measuring the PA signal amplitude with different light intensities and temperature;
2. Multiplying the coefficient of unit light intensity by the light intensities;
3. Multiplying the result of Step 2 by the difference between measured temperature and 36.5 °C;
4. The sum of measured amplitude and result of Step 3 is the compensated result.

**Figure 8:**
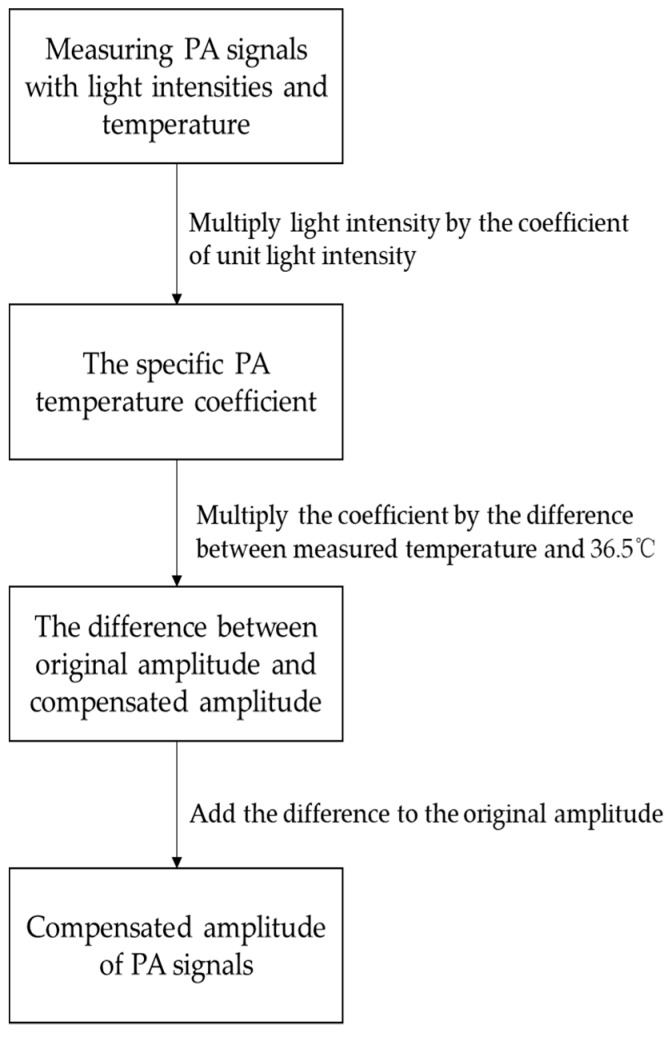
Flowchart of compensation.

The flowchart of compensation is as Figure 8.

## 5. Conclusions

In this paper, the relationship between the Grüneisen parameter and PA signal amplitude is introduced. The main factors in the Grüneisen parameter, the thermal expansion coefficient, sound velocity and specific heat capacity, are represented as functions of the temperature and concentrations. The theoretical results showed that the PA signal has a good linearity with the light intensity, glucose concentrations and temperature. This paper proposed a parameter, named the PA temperature coefficient, which can quantitatively represent the PA temperature characteristics. The PA temperature coefficient was only proportional to the light intensity. The PA temperature coefficient of unit light intensity was an approximate constant. This paper further conducted theoretical analyses by experiments using pure water and glucose solutions. The PA temperature coefficient by the normalized light intensity proposed in this paper will be a powerful tool for PA measurement temperature compensation. This conclusion can also be applied to PA measurements of other constituents in liquid.

## Figures and Tables

**Table 1:** Polynomial coefficient of the thermal expansion coefficient.

*i*	1	2	3
*x_i_*	1.0006	−4.9260 × 10^−5^	−4.0970 × 10^−6^
*y_i_*	3.9280 × 10^−3^	−4.8810 × 10^−7^	−2.6290 × 10^−8^
*z_i_*	−3.1670 × 10^−6^	1.8650 × 10^−8^	−1.4220 × 10^−10^

**Table 2:** Polynomial coefficient *b_ij_* of heat capacity.

*j*	*i*		
	1	2	3
0	5.7416	−1.0565 × 10^−2^	1.7381 × 10^−5^
1	−1.8569 × 10^−2^	9.2023 × 10^−5^	−1.3186 × 10^−7^
2	1.5461 × 10^−5^	−8.4373 × 10^−8^	1.2838 × 10^−10^

**Table 3:** PA temperature coefficient and light intensity.

Light Level	Temperature Coefficient (V/°C)	Light Intensity (V)
1	0.0458	1.6130
2	0.0676	1.8236
3	0.0986	2.1010
4	0.1235	2.5351
5	0.1179	2.9457
6	0.1323	3.4897
